# The Experiences of Parents of Children Undergoing Surgery for Congenital Heart Defects: A Holistic Model of Care

**DOI:** 10.3389/fpsyg.2019.02666

**Published:** 2019-11-27

**Authors:** Leeza David Vainberg, Amir Vardi, Rebecca Jacoby

**Affiliations:** ^1^Oncology Center, Assuta Hospital, Tel Aviv, Israel; ^2^Pediatric Cardiac Intensive Care, The Edmond and Lilly Safra Children’s Hospital, Sheba Medical Center, Ramat Gan, Israel; ^3^Medical Psychology Graduate Program, Stress, Hope and Cope Lab, Tel Aviv-Yaffo Academic College, Tel Aviv, Israel

**Keywords:** caregiving, CHD, ICU, parents, children, coping, family-centered care, surgery

## Abstract

The present article is based on a qualitative study focusing on parents of children born with congenital heart defects (CHDs) and hospitalized in the children’s intensive care unit post-surgery. Our aim was to explore parents’ subjective experiences as primary caregivers. Ten semi-structured interviews were conducted and analyzed using interpretative phenomenological analysis according to the instructions of Smith and Osborn. Our analysis yielded eight categories which were grouped into four themes and two main superordinate themes: (1) dialectical tension between positive and negative experiences; and (2) fluctuations between the inner and the outer world. The two superordinate themes intersect such that parents report positive as well as negative experiences within both their inner and outer worlds. Based on our analysis, we found that the experience of having a child undergo surgery for a CHD can be regarded as a chaotic period characterized by uncertainty, confusion, and helplessness. It is therefore no surprise that many parents display negative psychological outcomes which extend beyond the period of hospitalization and may also affect their future parenting and coping. However, within this chaotic and stressful situation, parents had occasional supportive experiences which decreased their emotional distress and isolation and helped them throughout this difficult period. We thus conclude that the support offered to parents during the hospitalization period should be increased by trying to minimize their negative experiences and strengthen their inner coping abilities. These changes cannot be implemented without also addressing the needs of the medical staff in their role as caregivers. Therefore, we propose a holistic model of care which supports both parents as caregivers of children undergoing surgery for CHD and the medical staff involved in their care.

## Introduction

Infants born with congenital heart defects (CHDs) nowadays are treated and often survive into adulthood (Tak and McCubbin, 2002). While these children undergo surgeries and recurring follow-ups, their emotional well-being is highly dependent on their parents’ psychological state and coping abilities (Arafa et al., 2008; Menahem et al., 2008a). Although parents are the primary caregivers and are often involved in their children’s care 24 hours a day, studies have, surprisingly, mainly focused on the psychological outcomes of the children undergoing surgery while ignoring parents’ experiences.

Congenital heart defect are heart ailments that exist at birth. They are structural or functional heart problems caused by a defect or abnormality and not by a disease (American Heart Association, 2017) which affects approximately 8 to 12 of every 1,000 live births and accounts for two thirds of all major birth defects (Chadha et al., 2001; Shah et al., 2008; Dolk et al., 2010; Hoffman, 2013). The reported incidence of CHD births has greatly increased over time, going from 0.6 per 1,000 live births in the 1930s to 9.1 per 1,000 live births after 1995 (van der Linde et al., 2011). Prevalence has been found to vary according to geographic location: for example, in the United States, CHD are the most common type of birth defect (Hoffman and Kaplan, 2002). In Israel, approximately 1.1% of all live births were CHD births between 2000 and 2006, meaning 11 per 1,000 live births (Agay-Shay et al., 2012 as cited in Agay-Shay et al., 2013). It has been suggested that variation in the prevalence of CHD worldwide is due, in part, to a lack of screening and to diagnosing the presence of defects rather than the absence of them. Such screenings, which are more readily available in high- and middle-income countries, have presumably led to the increase in the reported rate of live births in general as well (Shah et al., 2008; van der Linde et al., 2011).

Once an infant is diagnosed with CHD, a treatment plan is established and the parents and medical staff are exposed to ongoing challenges. While CHD was once a death sentence to children, the advancement of medicine and surgical techniques since the 1970s have led to a lower infant mortality rate (Hoffman et al., 2004). These improvements in medicine and surgical techniques have made CHD one of the most pervasive chronic pediatric illnesses, allowing many infants to be treated and to survive through adulthood (Tak and McCubbin, 2002; Daliento et al., 2006; van der Linde et al., 2011; Bajracharya and Shrestha, 2016). Currently, the infants with CHD mortality rate in upper-middle income countries lies between 3 and 7% and around 20% in low- and middle-income countries (Bernier et al., 2010). However, it is still difficult to estimate patient survival rates because patients are often operated and re-operated on later in life, at a time when surgical outcomes may be poorer (Hoffman et al., 2004), while mortality rates may be underreported in low- and middle-income countries (Bernier et al., 2010).

Children with CHD display many symptoms related to their condition: for example, failure to thrive (FTT) and developmental delay are evident in 86.9% of these children due to their difficulty ingesting food and expending energy (Shah et al., 2008; Marino et al., 2012). They undergo life-threatening and painful surgical procedures, require lifelong hospital follow-ups, and are at high risk of other chronic physiological issues such as infection and malnutrition (Menahem et al., 2008a). Nevertheless, Menahem et al. (2008a) showed that children with CHD who have undergone surgery display psychological functioning similar to control groups both before and after cardiac surgery. It was found that the best predicator of CHD children’s emotional well-being was their emotional well-being prior to surgery (Menahem et al., 2008a) and that parental emotional stability is a bigger factor in predicting children’s psychological well-being (Arafa et al., 2008).

It is only natural that researchers have tended to focus on the difficulties experienced by children with CHD. However, it is the parents of these children who, as their primary caregivers, carry the greater burden of care (Lawoko and Soares, 2002) and endure duress throughout the entire period of dealing with their child’s CHD (Tak and McCubbin, 2002). This is because the discovery of CHD often results in tension during pregnancy and medical interventions soon after birth. Parents encounter numerous challenges as they face doctor’s appointments, screenings, potentially fatal interventions such as open-heart surgery (Harvey et al., 2013), prolonged periods of hospitalization and care, multiple surgeries, developmental delays, near-death experiences, and death (Daliento et al., 2006; Helfricht et al., 2008; Shah et al., 2008; Marelli et al., 2014). This situation often becomes a difficult trial for the family unit which is providing medical and emotional care for their newborn child (Daliento et al., 2006; Menahem et al., 2008b). These parents experience greater financial instability due to unemployment, sick leave, and the medical costs incurred by taking care of their children – factors that lead to a decreased standard of living (Lawoko and Soares, 2006). They therefore report a lower quality of life (López et al., 2016) and difficulties in coping with everyday activities due to emotional distress (Alkan et al., 2017). This burden negatively affects parental psychosocial adjustment (Wray et al., 2018). It continues throughout the children’s life for mothers, while decreasing over time for fathers (Lawoko and Soares, 2002). Consequently, parents are at a higher risk of emotional distress, feelings of hopelessness, and suicidal ideation, with mothers showing higher levels of distress, anxiety, and social isolation than fathers in comparison to both depressed and non-depressed people (Lawoko and Soares, 2002, 2006; Woolf-King et al., 2017; Kaugars et al., 2018; Wray et al., 2018).

Prior to their children’s surgery, parents experience monumental stress (Wray and Sensky, 2004) as well as high anxiety, helplessness, lack of control, and emotional distress (Menahem et al., 2008b; Harvey et al., 2013; Woolf-King et al., 2017). While these studies have all shown that these symptoms persist post-surgery, others have found that parental anxiety levels return to baseline (Wray and Sensky, 2004; Menahem et al., 2008b). However, even when a child is successfully recovering post-surgery, as long as they remain hospitalized, parental stress has been found to persist at high levels (Heuer, 1992; Franck et al., 2010; Harvey et al., 2013). Additionally, it was found that parents of children undergoing heart surgery are at a greater risk of developing acute stress reaction (ASR) or acute stress disorder (ASD) (16–30%) and post-traumatic stress disorder (PTSD) (15–34%) or presenting with clinically significant symptoms of trauma (32–80%) (Farley et al., 2007; Helfricht et al., 2008; Landolt et al., 2011; Woolf-King et al., 2017). Mothers of children with CHD displayed more symptoms of PTSD than fathers (Wray et al., 2018). It is therefore clear that these parents’ psychological well-being is in danger (Lawoko and Soares, 2006), and that these psychological repercussions may lead to greater coping difficulties (Harvey et al., 2013).

As primary caregivers, parents of children with CHD have difficulty coping on yet another front: the medical system. In 1959 the Platt Committee, which consisted of both doctors and nurses, published the Platt Report, which brought the emotional needs of children as hospital patients to center stage by enabling almost unrestricted parental visitation (Platt, 1959; Davies, 2010). This was due to several factors. One was the changes in disease patterns, which resulted in a world where cancers, congenital defects, and what we now call chronic diseases were most prominent. These changes affected the care needs of children in hospital as well as those of the doctors caring for them (Platt, 1959; Davies, 2010; Taylor, 2015). Another factor was developments in the field of pediatric psychology that led to an understanding of the harm incurred by separating children from their primary caregivers at a young age. The Platt Report thus enacted changes that addressed, for the first time, the emotional and psychological needs of children. It did not, however, consider the needs of the parents providing them with care (Davies, 2010).

During the 20 years following the Platt Report, parents were tolerated by the nursing staff, who viewed them as visitors. Nurses were, in fact, expected to regard them as integral members of the children’s care team, but this transition was a threat to the traditional working practices of both doctors and nurses, who felt as if their own comfort and convenience were being ignored (Davies, 2010). This changed over time, and in the late twentieth century the focus shifted to family-centered care, which examined the needs of the entire family unit (Davies, 2010; Kuo et al., 2012). With the debut of family-centered care, pediatric nursing moved to a wider perspective which encouraged parental participation in childcare (Davies, 2010; Kuo et al., 2012), supplying parents with desired information and providing them with assurances and proximity to their children (Meert et al., 2013). These changes were instituted not only to aid babies and children in their psychosocial development but also to assist the psychological well-being of their family caregivers (Craig et al., 2015). Despite this shift, family-centered care has still not been sufficiently implemented (Kuo et al., 2012).

Few studies have referred to the experiences of parents of children with CHD with nurses or doctors in the intensive care unit (ICU). Heuer (1992) found that parental stress in the ICU remains high due to conflicting opinions by medical staff regarding their children’s health and care and the inability to remain in close proximity to their children. One study found that parents perceived nurses as supportive as they advocate for their children’s pain, provide emotional support, and include parents in the childcare (Gale et al., 2004), while another study (Kools et al., 1999) found that families of hospitalized adolescents with CHD were dissatisfied by nursing care due to contradicting expectations in care and communication. The disparities presented above resulted in parental hypervigilance in the ICU and both parents’ and nurses’ overall dissatisfaction. Another study found that fathers of children with CHD frequently felt overlooked and were only permitted to provide their child with peripheral care while in the medical setting (Gower et al., 2017). According to parents, members of the healthcare staff who are caring and cooperative and provided them with coping mechanisms and emotional support help them to feel more confident and optimistic about their children’s future (Wei et al., 2017). Additionally, Simeone et al. (2017) found that parental anxiety and stress decreased when provided with psycho-educational pre-operative interventions by the nursing staff. Nevertheless, the majority of publications build on nurses’ experiences in order to tailor parental assistance and care in the ICU and do not study the experiences of the parents themselves (Gavaghan and Carroll, 2002). Since it is parental satisfaction of hospital care and not solely the severity of their children’s disease that has been found a significant factor in parent’s psychological well-being (Lawoko and Soares, 2006), it is important to address this gap and develop this line of research.

In conclusion, parents of children with CHD have to combat difficulties on two main fronts; dealing with their children’s disease and process of treatment while simultaneously navigating through the medical system. These difficulties greatly impact their own well-being. The goal of the present study was to get closer to the experience of these parents as primary caregivers in order to gain a clearer perspective of their inner world and their experiences within the medical system. The idea for pursuing this line of research arose from the first author’s experience working as an interning psychologist in the children’s cardiac ICU where the study was conducted, after having witnessed firsthand the impact of the stress that parents accompanying their children in the ICU experience.

## Materials and Methods

### Participants

The current study includes 10 interviews conducted with 12 parents, two of whom were a married couple who chose to be interviewed together. The inclusion criteria for the study were participants who spoke Hebrew, were over the age of 18, and were accompanying a child or infant being treated in the children’s cardiac ICU in a children’s hospital in the center of Israel. A summary of the participants’ sociodemographic information is provided in Table 1.

**TABLE 1:** Summary of participants’ sociodemographic information.

**Participant’s name**	**Interview 1 – Rita**	**Interview 2 – Moshe**	**Interview 3 – Rachel**	**Interview 4 – Dana**	**Interview 5 – Avital**	**Interview 6 – Noa and Dan**	**Interview 7 – Daphna**	**Interview 8 – Reut**	**Interview 9 – Adi and Michael**	**Interview 10 – Yael**
Parent’s age	26	33	37	27	25	28	32	32	25	Unknown
Parent’s gender	Female	Male	Female	Female	Female	Female **and** male	Female	Female	Female **and** male	Female
Marital status	Married	Married	Married	Married	Married	Married	Married	Married	Married	Married
Educational level	Post high school	High school	Post high school	Undergraduate	Post high school	Undergraduate	High School	Undergraduate	Undergraduate	No Answer
Religiosity level	Very religious	Very religious	Very religious	Very religious	Very religious	Religious	Very religious	Secular	Religious	Very religious
Number of children	3	4	4	1	2	2	4	1	1	10
Child’s age	7 months	2 weeks	8 months	1 week	5 days	3 years	2 months	10 days	10 months	2 months
Child’s gender	Female	Male	Female	Female	Female	Male	Male	Male	Male	Female
Type of CHD	Hypoplpastic right heart	Hypoplastic left heart	No answer	Tetralogy of fallot	Narrow valve	Atrioventricular Canal	Hypoplastic left heart	Transposition of the great arteries	Blocked aorta	Cyanotic heart
Are future surgeries planned?	Yes	Yes	Yes	Maybe	No	No	Yes	No	No	No Answer

### Research Method

Qualitative analysis was chosen as the appropriate method for the present research. Qualitative research uncovers meaning by meticulously collecting large amounts of data and then extracting a story which aims to capture the complexities of life (Smart, 2010; Bondi, 2013). Qualitative health research (QHR) can be considered a subdiscipline of this field as it requires researchers to be not only well versed in the field of qualitative methodology but to have additional knowledge and skills including the ability to modify their methods through an understanding of staff roles, hospital codes, and patients’ physical and emotional condition. This allows the researcher to assess the clinical situation from different perspectives (Morse, 2010). Semi-structured interviews give parents the opportunity to express all aspects of their experience with the staff, creating a more honest and personal narrative of their impressions.

Several methods of qualitative analysis were considered for analyzing the interviews including: interpretative phenomenological analysis (IPA) (Smith et al., 1999), grounded theory (Glaser and Strauss, 1967), and narrative analysis (Clandinin and Connelly, 2000). IPA was seen to be highly relevant to the field of health psychology (Smith et al., 1999) because it aims to convey the participant’s own perception of an experience rather than present an objective account of it. It examines the participants trying to make sense of their own world and then tries to make sense of their process (Brocki and Wearden, 2006; Smith and Osborn, 2008; Smith, 2011) – a process which Smith (2011) calls “double hermeneutic” (Smith, 2011, p. 10). The narrative nature of the final analysis of IPA paired with its usefulness in portraying complexity and novelty were the reasons it was chosen for this study, enabling the creation of an analysis that goes beyond the standard thematic one (Smith and Osborn, 2003; Brocki and Wearden, 2006).

### Procedure

The first author approached parents with children hospitalized in the children’s cardiac ICU who met the inclusion criteria for the study in their children’s hospital rooms. She introduced herself by name and recited the following statement: “Hi, I have been working as a psychologist in this unit for the last year and am conducting a study with the support of Dr. X. I’m interested in studying the ‘journey’ parents undertake while dealing with this experience. If you have any time, I’d be happy to interview you.” If the parents responded positively, further information was provided, and the interview was conducted immediately. Interviews were conducted in one of three locations according to the parent’s preference: in the hospital room next to their child’s bed, in a private office, or in the empty family waiting room provided by the unit. The interviewer sought to respect the parents and their worldview and to be considerate of their time and needs within the hospital setting (e.g., tending to their child throughout the interview, answering phone calls, interacting with medical staff, etc.). Interviews lasted between 12 min and 1 h and 33 min and were recorded with the parent’s consent.

The interviews were conducted as semi-structured in-depth qualitative interviews according to the guidelines established by Smith and Osborn (2003, 2008) for use in IPA. Interviews were guided by a sequence of open-ended questions. Participants were initially asked to describe, in their own words, their experience in chronological order. Gentle prompts were used as the interviewer tried to complete pieces of the timeline or when she sensed that participants needed encouragement to provide more details or to speak more freely: for example, “Could you share with me how you felt?” The second part of the interview used the “funneling technique” (Smith and Osborn, 2008, p. 62) and focused on the parent’s experience within the medical setting via questions such as: “Can you tell me about your experience in the hospital?” “Could you describe your relationship with the staff?” As a final question, parents were asked if they had anything to tell parents embarking on the same “journey” with their child. All the participants received the same questions in the same order. Parents were given consent forms, which were administered before the interview began, and a socio-demographic characteristics questionnaire, which they filled out at the end of the interview session. The questionnaire included questions about education level, religion, family income, and parental age as well as information about the hospitalized child and their medical history. The questionnaires were optional and one parent chose not to fill them out.

### Ethics Statement

Ethical approval was obtained from both the hospital’s Helsinki committee and the ethics committee of the Academic College of Tel Aviv-Yaffo. Participants were informed that their participation would have no effect on their status at the hospital and that they could stop the interview process at any time. All the participants signed an informed consent form and were ensured that their anonymity would be maintained. At the end of the interview, parents were told that they were free to contact the researcher with any follow-up questions. All participants and medical staff were given pseudonyms to protect their privacy.

Two of the 10 interviews conducted were not fully completed. One parent was called to tend to her baby following a medical intervention, and one other requested completing the interview at a later time because her other children had arrived for a visit. Both parents stated that they did not want to withdraw from the study, but a later date was not arranged due to scheduling difficulties during their time in the ICU. Both only completed the initial, chronological telling of their experience, and this was included in the study. As the interviews, in general, progressed, the parental recounting of their experiences became repetitive, and it was therefore our impression that although these two interviews were not completed in their entirety, they were conducted sufficiently to accurately capture their perspective.

## Results

All interviews were transcribed by the interviewer and then analyzed by her and by another researcher according to the stages recommended for the IPA method (Smith and Osborn, 2003). The analysis had three phases. The first phase comprised reading the interviews and getting a holistic overview of the thoughts, feelings, and reactions that were expressed by the participants. The second phase involved extracting phrases or sentences from each interview and then grouping them into categories. The third phase was a comprehensive analysis of the categories designed to identify the key themes representing the various interviews. Each researcher did this stage separately and independently. Both researchers subsequently agreed that the chosen themes accurately represented the participants’ experiences. A closer examination of the data allowed for the creation of a more abstract description, which revealed two superordinate themes. The analysis ended with a higher level of integrative narrative complemented by an intensive discussion of the findings along with possible interpretations based on a comprehensive theoretical basis.

Several strategies were used to strengthen the plausibility of the results. First, a consultation with another qualitative researcher provided a comprehensive perspective on the data during all phases of the research. Second, the circular manner of analysis forced the authors to check for coherence between their interpretations and the narrative in a consistent manner. Third, direct quotes are presented so that readers can scrutinize the connections made between the data and the conclusions that were drawn (Shkedi, 2003).

### Holistic Perspective

The analysis revealed several layers of depth to the parental experience. The most noticeable aspect was that many of the participants were chaotic and disorganized in their speech and content, reflecting their stressful situation. The pace of the interviews was often fast at the beginning, with parents racing through their statements quickly and technically. In most of the interviews, parents described things as difficult without expressing their emotions, until they were gently prompted to expand on their feelings. The emotional distress, tension, and sadness were nonetheless palpable, even on entering the room. The parents’ body language conveyed anxiety and tension, and they often focused on every sound emitted by the machinery in the room, constantly cared for their sleeping children, moved restlessly, and cried or were almost brought to tears when recalling their experiences. As the interviews progressed, parents seemed to feel more comfortable to express their emotions, describe hardships, and disclose things that they deemed as more private. The interviews of parents who were able to verbalize their fears and emotional distress seemed to be more organized, even if the content itself was emotional.

### Themes

Our analysis produced eight categories: (1) parental emotional distress; (2) disrupted parental experience; (3) parental isolation and loneliness; (4) objectifying and critical medical staff; (5) parental resources and coping strategies; (6) parental involvement in childcare; (7) support system of family, friends, and each other; and (8) supportive, informative, and sensitive medical staff. The eight categories were grouped into four themes: inner negative experience; outer negative experience; inner positive experience; and outer positive experience.

#### Negative Experiences Within the Parents’ Inner World

##### Parental emotional distress

Parental emotional distress was present throughout the interviews. One of the most salient features was the uncertainty and helplessness pertaining to the entire experience, from the moment of initial diagnosis and throughout the child’s life. Fear, frustration, shock, and sadness were mentioned frequently as well as characteristics of the events themselves, such as the parents’ being exposed to vivid graphic imagery of their child, the speed at which events progressed, and near-death experiences.

*I was afraid, I was so very afraid. Out of fear*…. *I remember it like it just occurred, that I just, my whole body was shaking.*

(Rachel)

*It was*…*difficulty, tension, frustration, fear of what was going to happen. As time went by the tension heightened*…. *During that time, I remember carrying a lot of weight on my shoulders. I would cry to a friend. I need to just cry, to let it out, and let it out*.

(Daphna)

Parents feel helpless in the face of these events, as they have to entrust their child’s care to another person and have no control over the outcomes.

*That child is* [pause] *is my soul. It, he was in me and was inside of me, and* [long pause] *to suddenly give him to someone else who* [stops talking] *can fix him and I can’t? It’s a feeling of utter helplessness and* [long pause] *it’s because we as parents can’t do anything.*

(Adi)

They were shocked to receive the news of the initial diagnosis and had difficultly processing it.

We were in total shock of course, in the beginning. We didn’t know what these words were, what this defect in the baby even was and where it had come to us from.

(Reut)

They also reported the rapid unfolding events due to the risk involved.

*So they told me, it’s this hospital or that one. And then they told me*…*.”You’re moving to X hospital, and we’re already waiting for an answer from them, and the ambulance is on its way”*…. *She says to me, “quickly, quickly, pack up your things.” And again, I feel like they’re moving me, and I don’t know what’s going on all of the time.*

(Yael)

In addition, an internal conflict between their heightened emotions and their religious beliefs was apparent in parents’ reports.

*On the one hand, there is the strain of the thoughts I am having; on the other hand, there is my faith that* [trails off]…*There are ups and downs, and sometimes the fears, the thoughts, they get stronger and sometimes, you get stronger in your faith.*

(Moshe)

##### Disrupted parental experience

The birth of a child with CHD results in a disrupted parental experience. Parent–infant bonding and attachment is interrupted, parental fantasies are abandoned, and family routines and dynamics are uprooted, causing a period of loss, mourning, and adaptation. Additionally, parents seemed to struggle with the perceived identity of their child, comparing them to “normal,” “regular,” or “healthy” children. Thus, feelings of guilt and self-blame emerged in the interviews as parents grappled with their new reality.

All sorts of monsters that could come out [at birth] were described to me.

(Moshe)

We asked ourselves, why him, specifically. Where did this come from? Maybe I even blamed myself a little.

(Adi)

*Indirectly, yes, it’s because of me*…*It’s a heavy thought, it weighs on you. It’s not that I’m choosing to feel this way.*

(Moshe)

Many of the parents reported being separated from their babies almost immediately, rarely having the opportunity to bond, hold, and nurse them during their first weeks of life.

*He was in the preemie ward, and they took him straight away*… *I took him in my arms, only after three days.*

(Noa)

Parents mourned the experiences they were missing during their baby’s early stages of life.

*It was the only thing that I really felt during those times*…*everyone going to breastfeed except for me, everyone leaving the hospital with a baby carrier except for me. And then those are those specific moments that you just get hit with it straight in the face, it hits you that it’s a bit different and that you’re different from everyone else.*

(Dana)

The normal attachment process is often replaced by anxious parental behaviors. Parents often resisted leaving their children’s bedside, committing themselves entirely to their care even while they were unconscious, and neglected taking care of themselves during this time.

*I stand beside her most of the time. And I hardly sit at all*… *I barely make it to the bathroom*… *There is nothing else to do*… *I want to see her, I need to, I don’t know*… *I was with her 24/7 before, I’m very, very attached to her. That’s the reason I’m here all the time.*

(Rachel)

It makes me sad that I brought this child to the world, and I wouldn’t have let her be here alone like that for so many hours.

(Dana)

Parents also expressed worries about their children’s future alongside the desire to compensate them for their current experience.

I think about how she will deal with it later, however many surgeries she’ll need as she moves forwards in life and how much she will be able to be like all the other girls.

(Dana)

*And then I told him [my husband] that he has to make up everything the baby is lacking*…*the baby has been through more. I feel like he needs compensation.*

(Daphna)

Parents displayed tension between their desire to bond with their baby and their natural defense mechanisms for coping with the events that were transpiring.

*A week later, a week later they operated on her. It took me 4 days to get to her, my husband was* [trails off]. *My husband was here even during the surgery. I was not.*

(Rachel)

*I also didn’t know if he’d live or not*…. *But when you’re carrying the baby, you feel kicking, you get attached*…*that’s why at week 37 I said that I don’t want to hear anything about it until after the birth*…. *That’s how much I didn’t know if he would survive the birth.*

(Daphna)

#### Negative Experiences Within the Parents’ Outer World

##### Parental isolation and loneliness

Parents described incidents when family and friends were unsupportive, insensitive, and opinionated. Many parents also felt conflicted about sharing information about their child’s condition with other people or felt tension when they did share it due to the nature of the responses they received. These events resulted in loneliness and isolation for the parents and sometimes even from each other as a couple.

During that month when he was hospitalized, there was no one for me to speak to.

(Daphna)

*I don’t like to say too much*…*on the one hand I did want to tell people who asked me. At first I said, maybe I’ll hide it and I’ll say there are a few complications and we’ll be back from the hospital in a little while because she was born early.*

(Dana)

*You feel like it’s the biggest hardship you’ve ever experienced*… *They try to tell you that everything will be okay so that you won’t be depressed*… *You just want to be with your pain*… *you think only of it.*

(Avital)

*My mother could never see an ounce of blood*… *I didn’t bother to call her*… *The same goes for my family. They can’t stand that hospital smell. I have sisters who have never come here, never taken a shift for me here, for her.*

(Rachel)

Disagreements with their own parents also added strain to the parents’ lives, putting them under pressure to adhere to their rules and opinions.

*My mother was with me for that screening. Unfortunately, it made her hysterical her response was, what’s the problem, yes, have an abortion*…. *It’s frightening, the doctors are frightening. We said, there’s no way*…. *It was a very uncomfortable situation with a lot of arguments*…. *They told us it would destroy our home, that we should consider it.*

(Daphna)

*She [my mom] didn’t want me to come here and to see her with all of the things and everything [post surgery]. I actually wanted to come but my mom told me not to*…*it was hard, tiring* [begins to cry]. *You don’t know what’s going on, you get reports about how she is and all that, but you’re not with your child.*

(Avital)

Friends also voiced negative and insensitive opinions that left a lasting impression on the parents.

*They didn’t tell me unequivocally, it’s on you. But, in between the lines, from the things they said, it sounded like it. It’s apparently on my shoulders, why didn’t we have an abortion? Why am I letting him suffer? So I don’t talk to people from my work, for example. I know what they think*… *they think it’s cruelty.*

(Moshe)

Not all couples could rely on each other for support during this difficult time; some felt like they had to cope on their own.

*I couldn’t talk to him [her husband], he was broken, he had a hard time, even a type of depression I’d say*…*I couldn’t lean on him or trust him, it was hard to come to the hospital, and I couldn’t unload my feelings on him*…*he just couldn’t handle it.*

(Daphna)

##### Objectifying and critical medical staff

Parents spoke about negative experiences with the hospital staff, which created additional emotional distress as they navigated through the ICU. They reported on staff members being critical, objectifying, opinionated, and insensitive to their emotions or their personal beliefs.

*They told us horror stories, yeah, even when we were here for a talk. I did a screening*…*the doctor’s response was, “A mongoloid?,” just like that, in those words, in that tone of voice* [mimics him]… *that was his reaction. And of course we left the screening and we cried.*

(Dana)

*My mood rises and falls according to what a nurse or doctor says to me*…. *There was this nurse who said some bad things. He wasn’t supposed to say them*…*so as far as I was concerned he (the nurse) was dead to me, he said things he wasn’t supposed to say and they depressed me.*

(Rita)

Such interactions with staff members on whom they need to rely often created unnecessary strain.

*When a person is in such a state, both of us, young parents, it’s not the time to start asking why did you come here*… *just explain things as simply and encouragingly as possible, there is not much we can do about it.*

(Dana)

*Every time I see the same nurse who said that to me, I hope that she won’t be in our room because it would be very hard for me to receive help from her and to let her treat my baby*. (Dana)

There are times when parents’ beliefs conflicted with those of the medical personnel interacting with them.

*We arrived at the amniotic fluid test*…*and the results were abnormal. The doctor who performed the test responded with: “I know you trust the guy upstairs [i.e., God], but with these things you don’t play around, go and get an abortion.” It was important for everyone to tell me that he was an expert in his field and that he knew what he was talking about by like 90%, but we went with our faith and continued*…. *It wasn’t pleasant at all*…. *He gave us the information as is*…. *Of course, we left and cried afterward.*

(Daphna)

#### Positive Experiences Within the Parents’ Inner World

##### Parental resources and coping strategies

Parents of children with CHD who are hospitalized in the ICU after undergoing heart surgery are in the midst of a crisis and often function in a type of survival mode. Despite feeling emotionally distressed, they are required to maintain a certain level of functioning, care for the child who is hospitalized, and often look after their other children as well. The two most salient coping strategies emerging were religious beliefs and the search for meaning. These two strategies often appeared to go hand in hand.

You have to accept it and do what you need to do and deal with it the Lord gives strength.

(Moshe)

We were with our parents, praying. We read a lot of psalms.

(Adi on waiting during surgery)

We grew stronger in our faith, thank God.

(Michael)

Sometimes religion and the search for meaning were also construed as an opportunity for growth.

*The thing that helps me cope more than anything else, in these situations in life*… *is the inner belief in the Creator and the belief that everything He does is good. That there is a higher purpose that will eventually bring something good out of all of this that you have the strength to cope with this and that you will only grow from the experience in the end.*

(Moshe)

##### Parental involvement in childcare

Parents were positively impacted by their ability to have physical contact with their child in the ICU and to participate in their care. This helped repair the altered parenting experience and maintain a continuous parent–child relationship.

*I was happy to find out that I can do anything at all, that I don’t have to just sit there and watch*…. *It’s really important, and very good, because I’m coming from a place where I don’t know what I can and can’t do.*

(Dana)

Day to day, as a mom, I really am trying to do everything I can to make him [her baby] feel good, so that he feels that I’m really here for him, and here [in the ICU], it’s like I can’t do anything.

(Adi)

#### Positive Experiences Within the Parents’ Outer World

##### Support system of family, friends, and each other

Social support provided by family and friends had a positive impact on the parents as they struggled through the crisis. When family members were available to help with necessary tasks and were accepting and emotionally attentive, parents felt supported and reflected on these interactions positively.

*My mom came to spend the night here, so that my husband, who usually spends the nights here, could come home. My mom really helps; a ton*… *both of our family really rose to the challenge to help.*

(Dana)

Friends were also a great asset in terms of both emotional and physical support.

All our friends came together to help during the stage when he was hospitalized and we needed help with the kids. Really, our whole community, there wasn’t a single person who didn’t offer help.

(Daphna)

The couple’s mutual support also had a huge impact on their experience, enabling them to balance each other out during periods of difficulty.

Maybe it even connected us to each other a little.

(Adi)

Yes, maybe it did strengthen the relationship, when he wasn’t well.

(Michael)

When one supports the other.

(Adi)

You get through it together.

(Michael)

##### Supportive, informative, and sensitive medical staff

Interactions with the medical staff were perhaps the key influence on the parents’ experiences. Amid the stress of being in the ICU, parents felt relief when the medical personnel were professional, quick to respond to their questions, and emotionally sensitive. When they were supplied with medical information, parents found interactions encouraging and positive, and this, in turn, heightened their sense of agency, security, and control.

*And they also explain, they really explain everything*…*it really gives you a sense of control and understanding.*

(Adi)

*Professor Y. sat with us and explained to us very, very nicely and very clearly in a positive way. What had been explained to us previously had landed on us like a big mess, but he simplified things in the clearest way and told us what to do, what needs to be done*. *They provide care beyond their job description, they’re very nice and they encourage you to participate, for me to participate in what’s going on. It’s really important, and very good, because I’m coming from a place where I don’t know what I can and can’t do.*

(Dana)

Medical staff who did not objectify the parents and children were also seen to help tremendously:

*They really were very considerate nurses. They told me: “*W*hen you work with children and their parents it’s especially important*…*the baby isn’t an object, it’s a person and it doesn’t matter how small they are.”*

(Daphna)

*Every action they took they explained to us*…*they even had recommendations, like go and eat, drink, take care of yourself*…. *I think that what encouraged me was that they understood that I needed to preserve my strength.*

(Daphna)

### Analysis of the Results

Our results showed that within a largely negative experience, the parental experience of events, people, and emotions could be interpreted as both positive and negative. In and of themselves, these fluctuations could be confusing, and parents were found to vacillate between their inner and outer worlds. This led to the grouping of the themes into two superordinate themes. The first, the dialectical tension between positive and negative experiences, refers to the way in which parents perceived their experiences in the ICU. Parents displayed a wide range of feelings ranging from positive, constructive, and empowering experiences to negative, hurtful, and destructive experiences. The second superordinate theme is fluctuations between the inner and the outer world. This describes the vacillation that occurred as parents navigated between the rich emotional experiences of their inner world and their perception of the interactions with the outer world that surrounded and influenced them.

The two superordinate themes intersect so that there are inner and outer positive as well as negative experiences in both fields, as can be seen in Table 2.

**TABLE 2:** The two superordinate themes, four themes, and eight categories.

	**Dialectical tension between positive and negative experiences**		
		**Negative experiences**	**Positive experiences**
*Fluctuations between the inner and the outer world*	**Inner**	Parental emotional distress	Parental resources and coping strategies
		Disrupted parental experience	Parental involvement in childcare
	**Outer**	Parental isolation and loneliness	Support system of family, friends, and each other
		Objectifying and critical medical staff	Supportive, informative, and sensitive medical staff

To sum up, our results showed that parents experience a tremendous amount of turmoil while in the ICU with their children. There are, nonetheless, positive interactions that temporarily enable parents to shift from their negative experiences into more positive ones. The parents’ narrative was at times a strained, fragmented, and somewhat dichotomous view of the events transpiring around them. We thus observed their fluctuation between positive and negative experiences and between their inner and outer worlds.

## Discussion

The present study provides an in-depth analysis of parental experiences in the ICU while their child is undergoing surgery for CHD. According to our analysis, we found that the narrative of these parents is a disrupted timeline, characterized by uncertainty, confusion, and helplessness, much like the chaotic illness narrative as described by Frank (1995).

The negative aspects within the parents’ inner world found in our study are in line with previous research that demonstrated parents’ experiences as replete with stress, depression, and anxiety (e.g., Harvey et al., 2013; Woolf-King et al., 2017). Parents’ descriptions of shock, vivid imagery, fast pace of events, and the terror of almost losing their children are experiences that can lead to trauma (Shaw et al., 2006; Farley et al., 2007; Helfricht et al., 2008; Landolt et al., 2011; Woolf-King et al., 2017). We therefore suggest that the negative experiences observed in our study should be regarded as risk factors of ASR, ASD, and PTSD (World Health Organization [WHO], 1992; Nagata et al., 2008; American Psychological Association [APA], 2013).

Disrupted parental experiences occurred as parents attempted to adapt to a new and painful reality while parent–infant bonding and attachment were disturbed, parental fantasies of their newborns were abandoned, and family routines and dynamics were damaged. Previous findings similarly reported that stress is at its highest for mothers of newborns hospitalized in the ICU due to the alteration of their role as parents and the disruption to the initial parent–baby bonding (Daliento et al., 2006; Sikorova and Kucova, 2012; Diffin et al., 2016). Moreover, parents felt guilty, due perhaps to bearing witness to the perceived pain being suffered by their child and the stigmatization involved with the heart defect (Foster et al., 2001).

When it came to parents’ interactions with their outer world, our findings showed that parents were greatly impacted by the lack of perceived social support, which led to feelings of isolation and loneliness. Parents were in constant contact with the medical staff at the hospital and relied on them heavily. However, certain interactions with the medical staff increased parents’ emotional distress as they navigated their difficult experience in the ICU, reporting feeling objectified and criticized by staff who were opinionated and insensitive to their emotions and personal beliefs (see also Jacoby et al., 2018).

On the other hand, parents also experienced glimmers of positivity which provided them with a great sense of support and relief. In relation to their inner world, parents’ coping strategies relied heavily on their religious beliefs as a source of meaning and solace during their time in the ICU – a fact that can be partially attributed to the predominately religious population in this study. Religion has been found elsewhere to be used as a coping mechanism by parents of dying children (Hexem et al., 2011). Furthermore, religious coping has been found more effective than non-religious coping when the stressful circumstances are beyond one’s control (Wasserman et al., 2013). It might therefore have lessened the feelings of uncertainty, fear, and sadness that parents described as part of their experiences. Parents also benefited from being engaged in their children’s care – a finding which matches those of the Platt Report (Platt, 1959). This engagement is also a form of active coping as well as a partial remedy to the disrupted parental experience.

With regards to outer positive experiences, social support was highly appreciated by the parents interviewed. Social support has been proved an effective resource (LaMontagne and Pawlak, 1990) and a protective factor for patient’s health (Broadhead et al., 1983). Most important, however, were the interactions with the medical staff. When the staff acknowledged parents’ needs and did not refer to them as just extensions of their children, parents felt a great sense of relief and support. Such attention to their needs – such as the need for information, parental involvement, respect, and emotional support (Latour et al., 2008) – might therefore decrease their stress and improve their coping.

### Models of Interventions Within the ICU

In order to assimilate the above recommendations, attempts have been made to adapt the model of family-centered care to the reality of the ICU. One program, which lasted six sessions, aimed to assist parents in constructing meaning and processing their feelings about how their child is different from other children by using psychoeducation, encouraging maternal caretaking of the hospitalized child, and teaching caregivers active coping skills. The program was proved effective at lowering parental anxiety and worry while also improving the early mental development of the children through the mother–child care bond (McCusker et al., 2010). Another pilot program aimed to improve parent–nurse cooperation by increasing psychoeducation and participation in care and tailoring each step of parental involvement to the restrictions of the ICU itself. This resulted in an increase in parental satisfaction and self-efficacy and a decrease in parental anxiety and perceived anxiety regarding their “partnership” with the nursing staff (Uhm and Kim, 2019).

Attempts by nurses to engage parents in childcare seems to result in parents feeling more connected to their newborns, thus helping to counterbalance the disrupted parental experience described in our study and to begin repairing parents’ missed bonding experiences with their children (Gale et al., 2004; Beck et al., 2009; Sikorova and Kucova, 2012). Today, there are many disparities between the style and quality of care of different ICUs worldwide. Although it has been found that single family rooms within the ICU are superior for infant care and for parental satisfaction, that parental involvement in patient care is imperative, and that these factors influence both the length of the hospital stay as well as the chance of re-hospitalization, many hospitals have not created such conditions (Shahheidari and Homer, 2012). This is perhaps because the suggested programs concentrate on the needs of the parents but do not take into consideration the difficulties encountered by the medical staff.

### The Needs of the Medical Staff

Research has shown that high burnout scores and secondary traumatization are prevalent among pediatric nurses and nurses in the NICU due to the high stress and high volume conditions of these units alongside the exposure to the pain and helplessness of the patients and their families (Braithwaite, 2008; Profit et al., 2014; Tawfik et al., 2017). In the NICU and PICU, both doctors and nurses expressed concerning levels of PTSD symptomology (Dalia et al., 2013) and high burnout scores (Lazaridou et al., 2011). In addition, a meta-analysis by Low et al. (2019) found that doctor burnout is already prevalent during residency, and is especially high for surgical specialties. High burnout scores have been found related to low levels of job satisfaction and low personal well-being as well as high levels of emotional exhaustion, low morale, depersonalization, lack of meaning, absenteeism, and a low sense of personal accomplishment (Braithwaite, 2008; Meadors and Lamson, 2008; Profit et al., 2014; Kelly et al., 2015; Meyer et al., 2015; Barr, 2017; Tawfik et al., 2017). Compassion fatigue apparently mediates some of the association with burnout and low job satisfaction (Kelly et al., 2015; Meyer et al., 2015). These findings have, in addition, been found to effect the quality of health care personnel’s work; when not as exhausted, they become more emotionally engaged with their work and have a higher sense of well-being (Meadors and Lamson, 2008; Profit et al., 2014).

Similarly, when the medical staff are psychologically cared for, they care better for the needs of both the patients and families, exhibit more empathy, provide greater continuity of care, are less likely to make mistakes, engage better with other staff members, are less likely to leave their jobs, and assist in programs which aim to improve the unit (Meadors and Lamson, 2008; Profit et al., 2014). Social support at work seems to lessen the effects of burnout and secondary traumatization. Moreover, strong feelings of competency, reassurance of self-worth, and recognition at work lead to higher job satisfaction among nurses (Barr, 2017). It is therefore important to reinforce the resilience of health care personnel in difficult work environments such as the NICU. Research has also emphasized the need for prevention programs within these hospital units which focus on psychoeducation, increasing self-care, combating burnout and compassion fatigue, and teaching medical staff to identify and cope with the effects of traumatization (Braithwaite, 2008; Meadors and Lamson, 2008; Profit et al., 2014; Kelly et al., 2015; Meyer et al., 2015; Barr, 2017; Low et al., 2019). These programs, which foster social and psychological support for the medical staff, may be an effective tool against employee attrition and substandard medical care. While many studies have advocated for the integration of workshops and services catering to the emotional needs of nurses, there are few models of care for the medical staff as the patients’ formal caretakers within the medical system.

While our research has focused on parents as caregivers, the aforementioned studies have shown that it is no less important to consider the needs of the medical staff as formal caregivers – caregivers not only of the identified patient in the ICU but of the entire family. Following these studies, we found that parental experiences are strongly linked to the care provided by the medical staff. We thus believe that it is remiss to focus on only one side of the caregiving relationship and propose a holistic model of care that can be adapted into the framework of the ICU. Our model aims to consider the needs of all involved parties and implement improved working practices which will assist the patients, their families, and the caregiving staff by cultivating the interactions between them. As our current study’s primary focus was on the needs of the parents as the primary caregivers, interventions tailored to the needs of children in this setting were not included in the model.

### Our Approach: A Holistic Model of Care

Our holistic model of care comprises several general considerations. First, multidisciplinary teams including doctors, nurses, physiotherapists, psychologists, social workers, and parents should be involved in the treatment of the child and their parents. Second, the proposed interventions are most effectively implemented by psychologists and/or social workers trained and working within a hospital setting and possessing the expertise required to work with this population. Third, it should be noted that the integration of mental health care providers will ease the burden of care from the medical staff, who are often required to address the emotional distress of patients and their families without the proper tools, training, or resources to do so.

**FIGURE 1:**
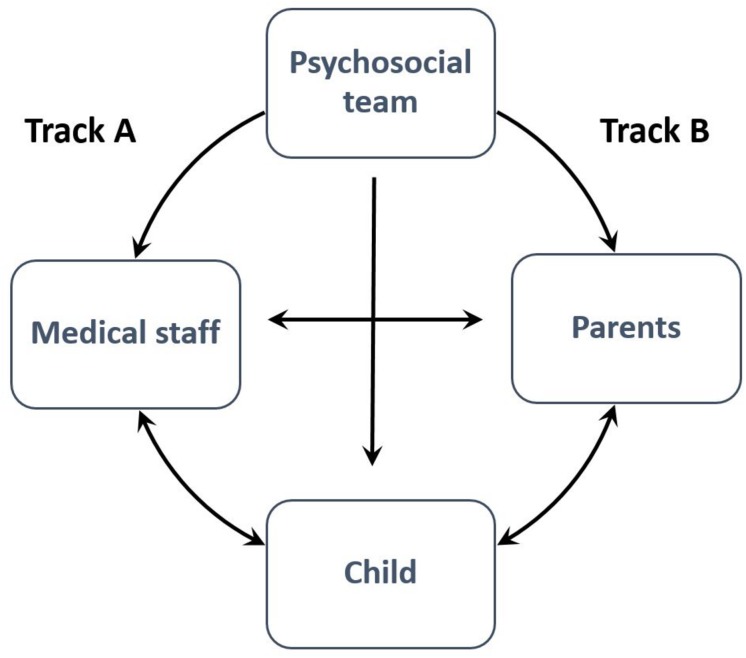
A proposed holistic model of care.

Our model proposes two tracks of interventions: Track A includes interventions aimed at helping the medical staff and Track B includes interventions aimed mainly at the patients’ parents’, i.e., the primary caregivers in the familial unit (see Figure 1).

Figure 1 presents a visual representation of the holistic model of care described above. The model features interactions between the psychosocial staff, the medical staff, and the parents, whose care is directed toward the child and their well-being. While the psychosocial staff greatly impacts the well-being of the individuals within the model, the interactions between them also improve in quality and well-being as a result.

#### Track A: Medical Staff

Track A consists of ongoing sessions provided to the medical staff with the psychosocial staff. Sessions can take place in larger forums which may include various hospital ward employees (i.e., doctors, nurses, medical secretaries, nursing assistants, etc.) or be held for a specialized audience (i.e., doctors only, nurses only). If the need arises, individual sessions should also be available to the staff.

The sessions will include: (1) early detection and/or self-assessment of burnout by standardized questionnaire or psychosocial interview; (2) psychoeducation about burnout, compassion fatigue, and self-efficacy at work; (3) training in communication skills (e.g., delivering information, breaking bad news, etc.); (4) encouraging and cultivating sensitivity toward parents’ needs. (5) Sharing experiences and interpersonal relationships between staff members (e.g., stress and loneliness as well as positive experiences); and (6) sharing and processing stressful/traumatic experiences (e.g., death of a child, aggressive behaviors, heightened parental emotions).

#### Track B: Parents

Track B includes the implementation of perioperative interventions with both parents, if possible, or with an individual parent. Interventions may also include other family members if necessary or creating an “open” support group. In some cases, meetings will also include medical staff members.

The sessions will include: (1) Biopsychosocial assessment; (2) psychological preparation before surgery including (a) providing information (psychoeducation), (b) stress management techniques, and (c) instructing the parents on how to prepare the child for surgery; (3) emotional support; and (4) encouraging the use of social support. Ideally, these interventions should take place during the time period between when the surgery is scheduled and the family’s arrival at the hospital. However, due to the realistic strain on hospital staff and the limited resources, we acknowledge that this may not be possible in most hospitals. This track also includes the provision of post-operative interventions when necessary. Post-operative parental emotional support is, accordingly, provided through two channels. First, the psychosocial staff member who met with the family originally will provide them with emotional support and instruct them on how to assist their child with emotional regulation, the rehabilitation process, and their return home. Second, the nursing staff will strengthen the parents’ active coping skills by instructing and supporting them through the initial care they provide for their child, as is possible within the constraints of ICU.

#### The Proposed Model

We believe that this approach will contribute to the well-being of all parties involved. It will improve the quality of life of the caregivers and consequently contribute to the quality of care provided to children in the ICU and help the children and their families have a better subjective experience overall. Combined with previous research in the field, this model addresses the needs of parents that emerged in this study together with the needs of the medical staff providing them with care.

## Conclusion

By conducting this qualitative study, we have given parents of children undergoing surgery for CHD a chance for their voices to be heard and their needs to be met. Our study demonstrates that the experience of having a child undergo surgery for CHD is tumultuous and inextricably linked to the parental experience of care provided by the medical staff. Consequently, the medical staff providing the families with care is, in turn, impacted by the children and their families. Our findings are supported by previous research about medical personnel in the children’s ICU.

We believe in a new model of care that can decrease the emotional distress and isolation that families experience during this time. We therefore propose a holistic model of care which can be implemented by the psychosocial staff within the ICU setting. We believe that even partial implementation of this model will lead to an improved emotional experience for both parents and children. The child should, in our opinion, be addressed as part of the family unit and not as a separate entity, just like the parents, as proposed in a previous study (Jacoby et al., 2018), should experience the illness together with their child as one “ill unit.”

Approaches to medical care are constantly evolving. It is, in our opinion, time for another leap – this time toward a more holistic approach to medicine and caregiving. It is important to take into account the needs of both the patients and their families and the medical staff, as well as the dyadic relationship between them. This integrative approach looks to ease the medical staff’s burden of care while improving the experiences of the patients and their families. Addressing the needs of both will inevitably increase the implementation of these practices, which will advance patient care in the NICU.

### Limitations and Future Research

This study was conducted using qualitative research methods. Qualitative research has limited generalizability due to its small sample sizes and can be biased, if not conducted carefully. It is true that the population in this study has particular characteristics, such as their religious beliefs as well as the culture of their country of residence. However, the in-depth analysis of qualitative data conducted in this study provides an important portrayal of the experiences of parents in this situation, which could not have been conveyed using quantitative research methods. Therefore, it was imperative to conduct this study using qualitative research methods in order to deeply examine a complex experience that has not garnered much research attention. Future studies could use quantitative research to supplement the model and assist in its implementation. Additionally, future research should provide a qualitative in-depth analysis of the experiences of the medical staff in similar settings, so as to further substantiate the proposed model.

## Data Availability Statement

The datasets generated for this study are available on request to the corresponding author.

## Ethics Statement

The studies involving human participants were reviewed and approved by the Ethics Committee at Sheba Medical Center, Ramat Gan, Israel. The patients/participants provided their written informed consent to participate in this study and for the publication of any indirectly identifiable data in the manuscript.

## Author Contributions

LD and RJ conceived and designed the study, analyzed and interpreted the data, drafted the manuscript, and critically revised the manuscript for important intellectual content. LD conducted the research. AV provided and formulated the medical information about the patients in this study.

## Conflict of Interest

The authors declare that the research was conducted in the absence of any commercial or financial relationships that could be construed as a potential conflict of interest.
